# Factors that may promote the learning of person-centred care: an ethnographic study of an implementation programme for healthcare professionals in a medical emergency ward in Sweden

**DOI:** 10.1007/s10459-018-09869-y

**Published:** 2019-01-10

**Authors:** L. Dellenborg, E. Wikström, A. Andersson Erichsen

**Affiliations:** 1 Institute of Health Care Sciences, Sahlgrenska Academy, University of Gothenburg, POB 457, 405 30 Gothenburg, Sweden; 2 Department of Business Administration, School of Business, Economics and Law, University of Gothenburg, Gothenburg, Sweden; 3 Sahlgrenska University Hospital, Gothenburg, Sweden

**Keywords:** Person-centred/person-centered care, Ethnography, Action learning, Implementation, Case study, Inter-professional relations, Ethics, Communication, Documentation, Disciplinary knowledge, Team, Management, Resistance, Context

## Abstract

While person-centred care has gained increasing prominence in recent decades as a goal for healthcare systems, mainstream implementation remains tentative and there is a lack of knowledge about how to develop person-centred care in practice. This study therefore aimed to explore what may be required in order for person-centred care programmes to be successful. The study used an ethnographic method of data collection. This consisted of closely following an implementation programme on a medical emergency ward in a Swedish hospital. Data consisted of participant observation and informal interviews with healthcare providers and their management leaders while they were in the process of training to use person-centred care. These interlocutors were using action learning methods under the guidance of facilitators. Our findings revealed that although the programme resulted in some of the processes that are central for person-centred care being developed, organisational factors and a lack of attention to ethics in the programme counteracted these positive effects. The study highlights the importance of facilitating mechanisms to produce desired results. These include management leaders’ learning about the dynamic and collective nature of learning processes and change. They also include allowing for inter-professional dialogue to enable managers and professionals to reflect deeply on professional boundaries, disciplinary knowledge and power relations in their teams. Teamwork is essential for the development of person-centred care and documentation, in accordance with this specific implementation programme, is also indispensable. The space for inter-professional dialogue should also accommodate their various perspectives on the aims of care and organizational reality.

## Introduction

For more than two decades, person-centred care has been upheld as essential for beneficial healthcare encounters (Stewart 1995; Mead and Bower 2000; Zhao et al. 2016). Influential actors, such as the World Health Organization (WHO 2000), the Health Foundation (2014) and the Institute of Medicine (IOM 2001) have highlighted the need for this approach to care. However, it is only in the last 10 years that researchers have been exploring how it may be implemented in the hospital setting (McCormack and McCance 2010; Bolster and Manias 2010; Ekman et al. 2011; Wolf 2012). A recent international review states that mainstream implementation of person-centred care remains tentative (Harding et al. 2015).

Person-centred care is an emerging area of knowledge. Most researchers acknowledge its humanistic and ethical value, although there is as yet no consensus about its definition (Edvardsson and Nay 2008). Commonly mentioned features include the importance of seeing the patient as a person with their own will, regardless of their physical or cognitive capacity. The person is considered free to act and take responsibility for making choices, family members are to be involved in care decisions, and decision making is to be conducted in partnership with the patient and, if possible, family members. This means examining power relations in the healthcare setting and how they influence the creation of a positive social environment. The healthcare practitioners’ emotional and interpersonal competences and the prioritization of relationships as much as care tasks must all be considered (Brooker 2004; Slater 2006; Edvardsson et al. 2008; McCormack and McCance 2010; Johnson and Abraham 2012; Røsvik et al. 2013; Ekman 2014; Björkman 2016; Zhao et al. 2016). The notion of person-centred care is often used interchangeably with others, such as “patient-centred”, “people-centred”, and “relationship-centred”, all of which lack clear definitions (McCormack et al. 2010; Zhao et al. 2016). In this study, we use the concept of *person*-*centred care* in order to stress the importance of changing focus in healthcare from the diagnosis of a patient to the person with a diagnosis (Ekman et al. 2011). We agree with Zhao et al. (2016: 400) that “a ‘patient’ is only a part, principally the physical and mental [sic] damaged parts, of an intact ‘person’.” In line with The Health Foundation (2014), we define person-centred care as an ethic that guides caregivers in their everyday work; it is not a model to be applied but is a fundamental philosophy of care that demands structural and organizational changes and strong leadership. This approach to healthcare influences the entire healthcare sector and requires the involvement of all healthcare stakeholders (Sjogren et al. 2012). However, the question of how this philosophy is to be integrated into regular care practice remains a challenge (Brooker 2012; Moore et al. 2017). There is a lack of knowledge about how to go about it and research results are only just beginning to emerge (Brooker 2012).

### Challenges to the implementation of complex innovations

Person-centred care differs widely from other types of implementation projects in healthcare. Rogers (2003) and others (Andersson et al. 2015), note that it is easier to implement innovations such as technical solutions, especially if these fall in line with what is considered to be “right” or “interesting” and support the current way of working. However, the way in which healthcare is organized tends to counteract efforts to introduce person-centred care (Wolf 2012), yet if patients are to benefit from its advantages, it must become part of daily practice. Implementation therefore requires cultural, structural and organizational changes (McCormack et al. 2010; Carlstrom and Ekman 2012; Wolf et al. 2012; Health Care Foundation 2014; Moore et al. 2017).

There is broad agreement that the facilitators of change need to understand and adapt implementation strategies to the characteristics of the innovation, the users, and the context (Damschroder et al. 2009; Eccles et al. 2005; Flottorp et al. 2013). However, implementation research lacks consensus regarding how the concept of context should be interpreted, how it is manifested and how contextual influences should be captured in research (Nilsen 2015). A common problem is that context tends to be referred to as a given constant, whereas in real life it is constantly shifting (Wikan 1992); it emerges and is dynamic and yet it is crucial to the phenomenon we are studying (Nicolini et al. 2003). Introducing a complex innovation in a complex environment needs to be explored carefully if we are to be able to provide practical guidelines for healthcare leaders and professionals (Craig et al. 2008; Moore et al. 2015).

This ethnographic study therefore aimed to describe and explore an implementation programme for person-centred care at the medical emergency ward of a Swedish hospital. We followed the implementation process closely in order to understand how healthcare providers responded to the implementation programme. The overarching aim was to gain a deeper understanding of learning processes in complex environment in which there are both barriers and facilitating factors.

## Background

In 2010, the hospital management of the medical emergency ward we studied decided that the care they provided should be person-centred. This decision was based on research that had shown that person-centred care can increase care quality, decrease the length of stay in hospital and be cost effective (Ekman et al. 2011, 2012; Olsson 2006, 2007, 2009). Using tested clinical guidelines and protocols, the management staff strove for quick implementation (Ekman et al. 2012). However, this proved unsuccessful and the management therefore had to consider methods of implementation (Abrahamsson 2014). They developed a programme in collaboration with organization consultants, educators and researchers (see Lindström Kjellberg and Hök 2014; see also Moore et al. 2017). It is the resulting programme that constitutes the subject of this ethnographic study.

### Description of the implementation programme

The programme, entitled “Personcentrerad vård i praktiken” (*Person*-*centred care in practice*) was developed collaboratively between the managerial staff at the hospital, the Gothenburg University School for Executive Education (GUSEE) and the University of Gothenburg Centre for Person-Centred Care (GPCC). It was led by facilitators, who coached a steering committee (*styrgrupp*) consisting of the medical emergency ward management leaders (a registered nurse (RN) and a senior physician), and some leading members of the healthcare providing team on the ward the managers had selected. The steering committee decided the goals of and dates on which the programme would be run. Table 1 shows the aims they agreed upon translated from Swedish into English by the authors.

**Table 1:** The hospital document with the steering committee’s aims of the implementation programme

*Effects on participants*
Deepened understanding of what person-centred care can be and how person-centred care may be implemented in clinical practice:
Each participant should be able to understand and reflect upon the concept of person-centred care
Each participant should be able to explain the concept of person-centred care and contribute to its implementation on the unit
A well-developed work method for planning and systematically implementing person-centred care for patients with different conditions and care needs:
Each participant should work according to agreed routines and goals for implementing person-centred care
Ability to enter into partnership with a patient and to formulate a care plan with a person-centred care perspective:
Each participant should have a clearly defined method for entering into a partnership with a patient
Each participant should be able to formulate a care plan based on the blueprints and instruments that have been agreed on in the ward
*Effects on the ward*
Consensus about the concept of person-centred care:
We stand united behind a systematic method for formulating care plans and working in a person-centred way
We have defined what an appropriate admission discussion should include
We work according to the ward round routines that have been agreed upon for each professional category
Having identified possibilities for and obstacles to introducing person-centred care in one’s own unit:
Everyone in the TEAM shares a common view of the benefits and potential following upon the introduction of PCC [person-centred care]
*Plan for continuing this work*
We must set up an action plan with clearly defined steps towards achieving our ultimate goal
We must decide how to describe how we aim to attain our goals
We must decide upon methods for measuring and following up on our goals
We must decide when we are going to carry out measurements
We must decide who is to carry out measurements and follow up

## Participants in the programme

The steering committee selected healthcare providers to participate in the programme based on their formal or informal leadership roles in the ward. Around 20% of the staff participated, and their task was to follow the programme and involve their colleagues in the process, mainly through learning-group activities. Involving non-participant staff was a key strategy for facilitating collective learning. There were 24 selected participants, consisting of three nurse assistants (NA), 8 registered nurses (RN) and 12 physicians (7 senior physicians and 5 registrars). Although the roles of the senior physicians and registrars (qualified physicians who are in specialist training) differ, they tend to increasingly overlap as the registrar becomes more experienced. The two management leaders participated alongside their employees. All of the participating nurses and the two managers were women; the majority of the participating physicians were men (two senior physicians and one of the registrars were women).

The facilitators consisted of a male consultant in organizational change, two healthcare science researchers and RNs who were specialized in person-centred care (a man and a woman). The facilitators’ task was to create a climate that encouraged cooperation, dialogue and enduring processes that helped attain the aims (cf. Rodgers 1971). On the first programme day, the two management leaders of the University of Gothenburg Centre for Person-Centred Care (GPCC) also participated—a female professor in healthcare sciences and a male senior professor in medicine.

During the implementation programme days, all participating nurses were present except for two RNs, who missed 1 and 2 days respectively due to workload and family reasons. Of the physicians, about half were present on the programme days, while those missing were mainly registrars, as seen in Table 2. The reasons given were work commitments from which they had not been granted leave. Only two of the physicians attended for all 5 days. The irregularity of the physicians’ participation was a major problem for the implementation programme.

**Table 2:** Participants present during the programme days according to profession

Programme day	RN (total 8)	NA (total 3)	Physicians (total 12, here divided into “senior physicians (7)/registrars (5)”)	Managers (total 2)
No 1	8	3	6 (5/1)	2
No 2	6	3	6 (4/2)	2
No 3	7	3	7 (5/2)	2
No 4	8	3	7 (5/2)	2
No 5	8	2	7 (4/3)	2

## The programme structure

The programme was organized into 5 intensive 8-h days that were spread over a period of 3 months from October 2013 to January 2014. They included an overnight retreat for days 2 and 3. Coached by the facilitators, the steering committee assessed each programme day and made detailed plans for the following one in order to follow the dynamic, changing context. The programme was based on seminars, lectures and workshops that were led by the facilitators. Learning group activities were carried out in the ward between programme days. The steering committee decided on the composition of the learning groups (*lärandegrupper*), and divided the participants into 6 smaller, inter-professional groups. The learning group activities (see Table 3) were designed to offer practical steps towards acting and thinking in terms of change, creating opportunities for inter-professional dialogue and raising awareness about the care environment. The overarching objective was to develop and implement person-centred care in the ward.

**Table 3:** Presentation of the learning group activities with instructions for the participants based on a hospital document written by the steering committee and the facilitators, translated from Swedish to English by the authors

Instructions for learning group activities		
Activity no 1: Set up a care plan together with a patient	Activity no 2: Organize lunch meetings with non-participating staff for discussions about person-centred care	Activity no 3: Organize a pilot project for implementation in the ward
Hold a healthcare provider-patient meeting to formulate a PCC [person-centred care] care plan/…/ Work according to the care plan and conduct the discharge meeting together. The starting point should be how the learning group collectively views PCC Describe how you went about formulating the care plan. What did you consider? What questions arose?	Invite some other staff from the ward to a lunch at which PCC will be discussed/…/	Building on discussions with other staff at lunch meetings, draw up a task for your learning group to implement. This assignment must further the development of PCC

## Methods and methodology used in the implementation programme

A key principle of the programme was that the power to make changes does not lie with the consultants and experts but with the staff of the workplace. In keeping with general action learning theory, the aim of the project was therefore to find solutions to problems in collaboration with those concerned and allow them to identify themselves, in learning groups, as agents of change in practice (Revans 1982, 1983; Gillett et al. 2017; Miller 2003). Accordingly, a fundamental assumption was that implementing person-centred care requires a programme based on person-centred methodology. Person-centred methodology means using both the participants’ expert knowledge of their work situation and the facilitators’ expert knowledge on social change and person-centred care to guide the implementation process.

The implementation programme promoted person-centred care as an ethic. This was based on a philosophical understanding of the person as being created through relations, as free to decide and act yet dependent on others (Smith 2010), and as possessing resources, strengths and abilities. The human condition is thus understood as one of both vulnerability and capability (Ricoeur 1992). The programme thereby both endorsed a particular philosophical perspective and contained theoretical propositions for putting this ethic into practice. It followed Ekman et al.’s (2011) proposal to initiate, integrate and safeguard person-centred care by documenting the patient’s narrative and enabling them to participate collaboratively in decision making through partnership. The programme simultaneously opposed top-down steering of the way in which person-centred care would be put into practice. It was left to the healthcare professionals and their management team to explore and develop their own methods while engaging in action learning methods in the learning group activities (see Table 3).

Importantly, resistance was seen by the facilitators as a crucial and anticipated element of change (Loup 2005). Time was therefore devoted in the group to exploring the notion of resistance and on discussing fears, hopes and expectations. Practical exercises, metaphors, animated illustrations and humour formed important elements of this pedagogical approach.

## Methodological considerations for the study

### Data production

In order to study this implementation programme, ethnographic fieldwork was conducted. This included participant observation (Nässén 2013; Savage 2000), informal interviews, natural conversations and analysis of documents. The researchers (LD, EW and AEA) were not involved in designing the programme and they played no role in the implementation process, with the exception of LD, who conducted participant observation. All three of the professional groups targeted by the programme–physicians, registered nurses (RN) and nurse assistants (NA)−were observed, as were the management leaders, the clinical top management leaders and the facilitators. The participants were observed on programme days as well as when they were engaging in learning-group activities on other days. Workplace meetings held in the ward during the period of the implementation programme were also observed and a follow up period of fieldwork was conducted after the conclusion of the programme (the programme ended in January 2014 but fieldwork continued until the end of 2016 and the results of this follow up are to be reported in another article). Observing the learning group activities required great flexibility on the part of the ethnographer since the group members had difficulty finding times when they were all available at the same time and often met up at short notice. It was therefore only possible to follow one learning group closely. All information about the methods used in the implementation programme was gathered through informal interviews with the facilitators, analysis of hospital documents and participant observation. Regrettably, the ethnographer was not invited to observe the steering committee’s meetings because the facilitators felt that these situations were too delicate to include an outside observer.

Participant observation requires meticulous, non-judgmental observation of interactions by the participating ethnographer. This helps them build an understanding of organizational and cultural behaviour from within. The method is suitable for exploring non-verbal features of everyday interactions and for noting discrepancies between what people say and what they do. This is invaluable in the study of complex, multi-professional settings such as a hospital clinic (Nässén 2013). Informal interviews and natural conversations are integral to participant observation (Savage 2000). They were essential in this study for capturing what was happening and how it was understood by the participants in an intensive, tightly-scheduled programme on a busy medical emergency ward. They enable the researcher to gain insights into people’s understandings of everyday events as they happen. Often, seemingly minor events may prove to be highly significant for the research question and they may relate to broader contextual issues (Eriksen 2009). Since this kind of event tends to be forgotten quickly, it is important to catch them as they happen. The researchers therefore paid close attention to apparently quotidien incidents. The method of participant observation thus enabled us to deepen our understanding of the way in which contextual factors were shaping relationships, actions and learning during the course of the implementation programme.

The facilitators, managers and participants collaborated in documenting the implementation process throughout. These documents were therefore also subjected to analysis.

### The methodology of ethnography

Ethnography is not simply a set of techniques for observing social phenomena but is also a reflective process in which the researcher strives to see beyond their own preconceived notions and beyond those of the participants (Wolcott 2008). It thus aims to yield a contextualized understanding of the world, seen from the point of view of the participants and it is based on both reflexivity and awareness of the interrelationship between historical, cultural and social factors. Describing human lives and understanding social phenomena in context requires that the researcher engages with the life worlds of others while remaining conscious of their own preconceptions—they must make clear how interpretation takes place (Skott et al. 2013; Scott-Jones and Watt 2010).

Ethnographic studies of organizations such as hospitals mean that researchers study people who occupy similar educational and social positions as themselves. Studying people in positions of relative power, challenges assumptions about anthropologists’ relationships with research participants, and demands that we reconsider issues relating to access, methodology, attitudes and ethics (Nader 1974: 301; Garsten and Nyqvist 2013: 14). In a hospital context, where strong professional identities and boundaries exist (Wolf et al. 2012), LD had to reflect on methodology, attitudes and ethics, and use her personality and role as researcher flexibly in order to gain access to and build relationships of trust with the physicians, RNs, NAs and management leaders. In her earlier fieldwork on this medical emergency ward, LD had earned the trust of the nurses (RNs and NAs) by downplaying her identity as researcher and helping out with care work. By contrast, when relating to the physicians, she had found that highlighting her identity as a researcher with a clear methodology and research aim was essential in earning their respect and making her role intelligible to them (Dellenborg 2013).

The “trust capital” that LD had earned earlier on proved to be invaluable in this study of the implementation of person-centred care, particularly in relation to the physicians, who were suspicious of the management leaders’ agenda. In order to maintain the physicians’ trust, LD found it necessary to distinguish herself from those involved in the implementation programme and make it clear that she was an independent researcher.

### Data analysis

Data analysis was conducted by the first author (LD) but regular discussions were held with the co-authors (EW and AEA) concerning analysis, interpretation and methodological issues. LD had taken fieldnotes by jotting down keywords while conducting participant observations and later in the day filling these out with detailed descriptions and narratives. The resulting field data consisted of notes from observations and informal interviews with programme participants, management leaders and facilitators. In ethnography, description and interpretation are understood to be intertwined through a hermeneutic circle. The researcher translates and interprets as they observe, and analysis begins with the process of writing up fieldnotes (Clifford 1986; Skott 2013). Both the jotting down of notes and the writing up of detailed narratives involved constant personal reflections upon insights gained (Roper and Shapira 2000). The analytical approach used largely followed that of Roper and Shapira (2000) and Eriksen (2009). LD read her notes many times during and after fieldwork, and she picked out and categorized issues that recurred and that she felt deserved further attention. “Frequently, the use of one’s observations becomes clear only when one sits down with one’s thick bundles of notes, trying to discover or impose patterns, regularities and interconnections in one’s often sprawling material” (Eriksen 2009: 45). When analyzing bundles of notes from cumulative field data, patterns emerge as significant and interesting to pursue further (Eriksen 2009). Gradually, such patterns develop into theoretical propositions. This process is not linear—the researcher weaves back and forth between the specifics of the data and generalizable theorems (Roper and Shapira 2000). Ethnographic knowledge thus typically arises out of an iterative hermeneutic process as the ethnographer oscillates between closeness to the field, during participant observation, and distance, while writing and reflecting (Geertz 1973; Skott 2013). This process is described as an hermeneutic circle. Understanding grows in ever-widening concentric circles as the researcher shifts focus from the parts to the whole and back again, in a reflexive questioning of their own pre-understandings (Gadamer 1989).

This reflexive stance is crucial to the rigour of ethnographic study. The researcher continuously interrogates their own presuppositions and tests them against the interpretations of the research participants and co-authors. Triangulation was effected using informal interviews and natural conversations with representatives of all the professions, and each revealed their various perspectives. Regular discussions with the facilitator, who read and commented on anonymized fieldnotes, were also important.

### The researchers’ background and skills

The first author is a social anthropologist who has extensive experience of conducting ethnographic fieldwork in Senegal (Dellenborg 2004, 2007, 2009) as well as in various healthcare settings in Sweden (Dellenborg et al. 2012; Wolf et al. 2012; Skott et al. 2013; Erichsen Andersson et al. 2018; Dellenborg and Lepp 2018). The second author (EW) is a researcher who has extensive experience of organization studies and implementation research in the healthcare sector (Grill et al. 2014; Arman et al. 2014; Wigert and Wikström 2014; Grill et al. 2015; Liff and Wikström 2015). The third author (AEA) is a researcher who has specialized in implementation research in hospital settings (Andersson et al. 2012, 2014, 2015; Erichsen Andersson et al. 2015, 2018).

### Ethical considerations

The project has been approved by the Regional Ethical Review Board in Gothenburg, Sweden (ref. 470-14). Participants were given information in accordance with the four principal requirements of the Declaration of Helsinki: autonomy, non-malfeasance, beneficence and justice. LD received written consent to participate from the management leaders and oral consent from the participants. The nurses were informed at several staff meetings and the physicians in individual phone calls, since they were scattered around the department and rarely gathered in a single meeting. Participant observation demands considerable ethical awareness on the part of the researcher and transparency about the study and the researchers’ role. It may be difficult for participants to express uneasiness about participating in the study, and the ethnographer must be sensitive to this. In this study, only one person refused to grant consent. The ethnographer therefore agreed to take no notes about anything this person did or said and she made an effort to respect the person’s personal space on programme days when they were present.

### Study context

The implementation programme was run in conference centres outside of the hospital except for the fourth day, when it was held in the hospital area. The healthcare workers and management leaders in this study all worked on a medical emergency ward at a hospital in western Sweden. It comprises an intensive care unit and a post-acute unit. At the time of this study, there were 35 beds in total. The average length of stay for patients was 4.4 days. The most common diagnoses were atrial fibrillation, myocardial infarction and chest pain. The treatment regimen included full supervision of the patients and readiness for emergency intervention by the personnel. High numbers of patient admissions and discharges, daily rotation of staff and high turnover rates of different care professionals and students meant the ward’s agenda shifted rapidly. The environment was high-tech, and a biomedical perspective was prioritized while care-giving was generally given less attention and was left mainly to the NAs (Wolf et al. 2012). The staff consisted of 60 RNs, 40 NAs and 43 physicians, of whom 14 were senior physicians and 29 were registrars, most of whom were in specialist training. This meant they were only present at the department intermittently. About 40% of the physicians and 75% of the nurses were women. The majority of the NAs were women.

## Findings and reflections

The findings are presented in two parts. The first gives a short description of how implementation projects have generally been conducted and received in this ward prior to this study. The objective here is to put this implementation project into a brief historical context. Although the time span is only a few years (2009–2016) it is revealing about the culture of the ward and the management’s understanding of how change is achieved. The second part describes a number of significant features of the implementation of person-centred care.

### Part 1: An historical perspective 2009–2016

Several implementation projects were carried out in this ward during the first author’s (LD) ethnographic fieldwork periods 2009–2012 and 2014–2016. These were the outcome of top-down decision making. They had been drawn up by politicians, the National Board of Health and Welfare or the top level clinic managers. The programme for person-centred care that formed the object of this study had also been designed by top-down decision making but it differed significantly from previous ones because of its emphasis on involvement, participation and iterative processes. Earlier initiatives had typically been led by a few healthcare workers who were selected by the management leaders and given some training that could be anything between a couple of hours to a couple of days long. They were then tasked with training and supporting the rest of the staff in working according to the new guidelines. These were characteristically described in terms of aims and reminders were announced at staff meetings and hung on the walls of offices in the ward. The staff tended not to be involved in the planning or initiation of these projects but they were simply given information about them at staff meetings, when they were given a short time to discuss them. The projects aimed to achieve changes in organization, such as prioritizing of patients, documentation systems, in-hospital medication reviews, ward rounds, clothing and hygiene routines and care procedures. The first effort to implement person-centred care mentioned in the introduction is a further example.

The ethnographer noted that these earlier projects were, without exception, met with resistance by the professionals affected. Only new routines that were essential in order for a staff member to be able to perform their job were adopted. One example was a new documentation programme that the RNs had to use in order to be able to communicate with the municipal authorities about the care of discharged patients. However, whenever it was not essential to adopt a new routine, staff tended to continue using the methods they were familiar with. A pertinent example is various changes made to the ward round routines. The purposes of the new routines were generally understood differently by the various groups and consequently, they were implemented by some and not others. This caused confusion and frustration concerning roles and responsibilities within the team. As we shall see, the historical background described above influenced the way the participants and the management leaders responded to the implementation of person-centred care.

### Part 2: The implementation programme for person-centred care

In this study, we found three main features of the implementation programme for person-centred care: (1) local challenges described by the participants, (2) barriers to collective learning and implementation and (3) effects of the programme (see Table 4). We describe these in detail in the following sections.

**Table 4:** Significant features of the implementation programme

Local challenges to person-centred care according to the participants	Barriers to collective learning on and implementation of person-centred care		Effects of the programme			
Organizational factors Team behaviour Differing understandings of person-centred care according to profession	Physicians’ lack of involvement in the implementation process	Physicians’ lack of confidence in the management leaders	Development of a common goal and an evolving feeling of a “We”	Insights into communication patterns that silenced patients’ perspectives	Improved inter-professional dialogue	A documentation-oriented focus on care

## Local challenges described by the participants

During the first day of the programme, the participants were asked to reflect on challenges to person-centred care in their ward. All three professional groups described challenges relating to organization, such as the lack of an efficient care chain, a heavy workload and discontinuities in the team due to individual scheduling. They all questioned the feasibility of implementing person-centred care when the staff were already overburdened. The facilitator remarked to the staff that they have to accept that some things cannot be changed, and that they:

need to work as guerillas and develop within the framework of the present [system]… To be able to really work in a person-centred manner, you would need to change even the architecture of the hospital/…/Under the current arrangements, there are certain premises that you have to accept, that you cannot change – and others that you can change.

The healthcare staff also saw team behaviour as a challenge. They said that patients and their relatives were sometimes not consulted about care and there was a lack of effective communication and teamwork on the ward. The nurses admitted that they needed to give patients more opportunities to be active, for instance by letting them serve themselves at mealtimes. A particular challenge that the RNs and NAs noted was the way the different professional groups understood what person-centred care means. They were concerned that it would be difficult to convince the physicians of the value of patients’ active participation in their care and of working as a team. They feared that this would mean the programme would result in “a half-implementation of person-centred care”.

These differences in understanding became evident when the physicians and the nurses expressed different attitudes towards the aim of the programme. The physicians questioned the value of the programme given the broader context of healthcare policy that they believed was detrimental to good care. They were unanimous in their scepticism towards the possibility of working in a person-centred way while the healthcare system was suffering from economic cutbacks, shorter inpatient treatment periods and a lack of continuity in care. By contrast, the nurses focused more on what was possible under given circumstances. Although they expressed frustration about the situation in the ward with heavy workload and discontinuities in the team, the nurses were convinced of the importance of providing person-centred care regardless of the context, and they were curious about how it differed from what they were already doing.

## Barriers to collective learning and implementation

### Lack of involvement by the physicians

Two major barriers to the motivation to learn about and implement person-centred care were found. The most significant was the difference between physicians’ and nurses’ attitudes to involvement in the programme that became apparent as it got under way. In informal interviews, physicians said they had not been involved in deciding about the programme and they felt pressured to attend the meetings. Their working conditions did not allow for this and they therefore had to squeeze programme days into their already full schedules. This critique remained informal. By contrast, the RNs and NAs had decided to participate in the programme long before it started, and their work schedules had been adjusted accordingly. The physicians also said they were tired of projects like this whose outcomes were never evaluated and suspicious of the fact that the presumed benefits of person-centred care had never been “scientifically verified”. They questioned whether indeed the supposed need for person-centred care was rooted in research or among patients. Nevertheless, some physicians did express an interest in learning more about it. For instance, one senior physician pondered:

the whole discussion about ‘person – patient’ has meant that one has more respect. For example, when it comes to times for examinations … [and I] avoid discharging anyone before lunch when we know that it won’t get done … respect for people’s time.

However, it was clear that the physicians’ lack of commitment to it mitigated against implementation. The physician quoted above was irritated about being pressured to attend the programme days and said he wished the management had at least involved him and his colleagues in some sort of motivating discussions before the programme started. He eventually chose to respond with a certain passive resistance:

I chose to see the generic ideas [as communicated in the programme] as something valuable; this issue of change and how it occurs, where the impulses come from, how you get people to participate. [I] chose to look beyond the specific issue of person-centredness.

Although the nurses were keen to take on the challenge of implementing person-centred care, they kept a low profile during the first three programme days. They were noticeably sidelined by the physicians’ frustrations, which occupied most of the time. After the third day of criticism by the physicians, the facilitators recommended that the ward management leaders postpone the programme. However, with the support of the clinic’s top management, the managers decided to continue nonetheless.

### Physicians’ lack of confidence in the management leadership

The other major barrier to implementation was the physicians’ lack of confidence in the management leaders. This was revealed in informal discussions, when they complained of not having been involved in the process, and in their overt criticism of local and national healthcare policy. Their main suspicion was that the management was trying to reduce the number of inpatient days for patients instead of promoting patients’ participation in their care. It had been noted at this hospital that wards that were following the programme’s guidelines had shortened care times per patient, but this had led to a higher patient turnover rate and, consequently, an increased workload foremost for registrars and RNs. The registrars in particular therefore expressed their concerns that the management would not compensate them for the extra time they required to follow the guidelines. One of the registrars said:

I’m not negative, it’s just that I want to know if [this change] will be covered by the existing budget … Have we got the support of the administration for this? … During a transition period we need to invest time./…/We can’t even keep our heads above water [as it is].

The registrars had calculated that the cost of running the programme was equivalent to one registrar’s salary for six months, and argued that employing another registrar would have been a better way to invest in good care than running this programme. They warned that patient safety was at risk:

If I discharge more than three patients after lunch, patient safety, quality and person-centredness will be at risk … If I could see anything [in this way of working] that would ease my situation, such as for instance having the time to go to the toilet…

Later on in the programme, an incongruity between the management’s and physicians’ notions of good care emerged that seem to confirm the physicians’ suspicions. The clinic top management leader was invited to listen to the participants’ presentation of their learning group activities. Two learning groups had tested working with new ward round routines that they had developed for a common care plan (see Table 3 on learning-group activities). The registrar reported:

The care plan gave rise to a better flow [of patients], more discharges/…/For the whole enterprise, the patients and staff it was better, but as a registrar, I had to deal with many more discharges. It was too much - I’m not prepared to do this!

The clinic management leader commented that wards that were practising person-centred care had shortened hospitalization time by an average of 1 day per patient. The turnover of patients had thereby increased by 20%, which may be burdensome for the staff but is a success for the clinic. He continued, talking in abstract terms of “better flow”:

Beds that become vacant are immediately filled again. It’s heavy for those starting out, but if everyone makes an effort to vacate beds and get them filled again then this will even out throughout the building. You have to be tolerant about more beds being vacated in one place than another on certain days.

Person-centred care, he said, was a way to increase efficiency and “lower the number of occupied beds”. He also called for “solidarity between colleagues at the clinic”. Given that the physicians had said they were suspicious of the management’s agenda as simply an attempt to reduce the length of hospital stays, it was remarkable to see that they remained silent when the management leader said this. And although the registrar had expressed despair about untenable working conditions, none of his colleagues or other participants now spoke up in his defence.

## Effects of the programme

### The development of a common goal: an evolving feeling of an inter-professional “We”

Progressively during the programme, the physicians fell into two groups. One remained critical to the implementation programme, while the other became engaged in collective learning to alter care routines. The latter was, notably, engaged in the learning group activities. Some of those who had questioned the programme most heavily actually became leading figures together with a group of nurses in developing routines for the common care plan. A significant change in the physicians’ attitude was discernible on the penultimate programme day. This was the first occasion on which the physicians did not voice criticism. Remarkably, a registrar who was participating for the first time was met with silence from his colleagues when he questioned the rationale of the programme and talked about a lack of time. It was as though the group of physicians had passed this stage of resistance. Instead of arguing against the programme, the physicians now cooperated with colleagues, RNs and NAs and actively engaged in improving their learning group projects. One RN, who had missed the second and the third programme days and overnight retreat, was astonished by what happened on this fourth programme day. She announced to everyone how the first programme day had been marked by defensiveness and an “Us & Them” feeling, while now there was “this feeling of Us!” In an informal interview, a NA also commented on this change and enthusiastically exclaimed:

Something’s happening to the communication here! Now we’re just Us, and not Us & Them.

Another RN suggested that the learning group activities were the circumstances triggering this process:

None of us had known [what to do], but we were creating something new together [in the learning groups] – [this] gave rise to a feeling of ‘us & ours’.

Although the physicians did not talk in these terms, their engagement in the intensive inter-professional cooperation was apparent. Clearly, the learning activities and the overnight retreat had an effect. Long after the programme had ended, one of the RNs said that the retreat had given them an opportunity to reflect and exchange ideas about difficult aspects of working on the ward, such as “hierarchical relations between professions and conflicts concerning routines”. Although the tone between the participants was generally collegial, the delicacy of these encounters is illustrated by the following conversation that took place after a workshop during the retreat:

Registrar (with resignation): “as a physician one has to defend oneself … the nurses are angry with the physicians.”

NA (emphatically): “yes … all this …mudslinging is unfortunate.”

Registrar: “Yeah, mudslinging … I can’t cope … it would be better not to meet …”

### Improved inter-professional dialogue

The above citation is a typical example of the complexity of relations between nurses and physicians and the way in which they can lead to frustration and unresolved misunderstandings in the team (Dellenborg 2013; Dellenborg and Lepp 2018). The ethos of this ward was characterized by a focus on tasks and the privileging of biomedical and technical knowledge over care knowledge (Wolf et al. 2012). The tension between different professionals and their types of knowledge was particularly evident in the organization of time and space during the ward round—the physicians did most of the talking and tended to sit with their backs to the nurses, and this hindered inter-professional communication (Dellenborg 2013). It was therefore encouraging to see how communication between the participants in the programme improved during the implementation process.

One particularly significant observation concerned a learning group meeting on the ward, at which the participants set up a care plan together with a patient (Learning group activity no 1, see Table 3). The usual ward round routine was abandoned and the RN, the NA and the senior physician gathered informally around a table in the team’s office. Having read the patient’s notes and listened to a report by the nurse in charge of the patient, they began reflecting on the patient’s problems. All three actively engaged in considering the care plan, giving one another equal space to speak and be listened to. This was the first time the observer had ever witnessed something like this taking place during a ward round at this department. Although this new communication pattern was not consistent throughout the programme, the learning group activities definitely gave rise to moments of inter-professional dialogue. As noted, the nurses commented on this and described how a feeling of “We” was evolving. However, neither the factors that contributed to greater inter-professional communication nor methods to sustain it once the programme had ended were mentioned by either the participants or the facilitators.

### Insights into communication patterns that silence patients

While the issue of inter-professional communication was not openly discussed, the issue of communication with patients was. During the second and third programme days during the overnight retreat, the participants reflected on their experiences of setting up a care plan together with a patient. They noted how certain communication patterns eclipse the patient’s perspective. They talked about the importance of allowing the patient time to pose various kinds of questions. They also said they realized that patients found it difficult to formulate care goals in response to direct questions and that dialogue was needed to support the patient in formulating realistic goals. These insights prompted the participants to suggest ways to communicate based on dialogue. One learning group reported on their experience of setting up the care plan, in which the problem identified by the carers turned out to be completely at odds with the patient’s experience. The senior physician and RN involved were astonished by this incongruence and they self-critically examined the way they had simply taken their own interpretation as given and had therefore initially failed to pose the questions that they subsequently realized would have enabled the patient to describe their own experience of his situation. The senior physician explained:

We don’t usually ask ‘what do you expect from care’ and we don’t give them time to answer/…/when we do, we get different answers than we’d expected.

Another senior physician interpreted this as person-centred care:

What’s good about person-centred care is that you can understand each person’s expectations.

The participants also reflected on the fact that the patient had little chance to play an active role in decisions made about their care when they were presented with a ready-made plan. In informal interviews, the nurses reflected on how their relationships to patients differed from that of the physicians because of the professional hierarchy. They felt it would be more difficult for a patient to question or alter a care plan if it was presented by a physician rather than by a nurse and that this would make it harder to develop shared decision making routines. The nurses said that despite the fact that they were based on decisions made as a team and involving the patient, care plans written by physicians in the learning group were essentially simply medical treatment plans, whereas the nurses included a caring perspective. The nurses expressed ambivalence about this. On the one hand, they wanted the physicians to “start” the care plan, to be actively involved in writing it and to lead the conversation with the patients on the ward round, but on the other hand, they saw problems with this. However, although these issues were raised in informal conversations, they were not openly aired during programme meetings and private discussions about communication patterns did not evolve into open debate about inter-professional communication.

### A documentation oriented focus on care

One effect of the implementation programme that we found negative was its narrow focus on documentation and routines, and its lack of attention to a person-centred ethic. From the very first day of the programme, the participants expressed concerns about person-centred care meaning more documentation and administrative chores and they wondered what other tasks would have to be left unattended to. They were occupied with questions about content and labour division—who should document what and where in the records. In particular, the question of who should be responsible for opening the care plan in the records system and start writing was tossed back and forth between the physicians and the RNs. The physicians largely thought it should be the responsibility of the RNs while the RNs argued that the physicians should do it so that they would be actively involved in the care plan and thus improve teamwork. The physicians were concerned about documentation being duplicated and at first, they saw no need for this common care plan since they kept their own daily records of the patients’ medical treatment plans.

One thing that did however motivate the physicians to work with common care plans was the finding in one of the learning group activities that the documentation of medical treatment plans was in fact often inadequate or non-existent. The team members had not previously discussed this problem with one another and it had therefore not been recognized at an organizational level, but it had affected individual patients and nurses greatly. Nurses often had to respond to the patients’ questions about altered plans when a new senior physician took over each Monday. During participant observation it had also been noted that the lack of documentation meant physicians often had to call the colleague who had been responsible the week before to ask them about the plan or simply start a new one. The physicians saw that this led to significant work duplication, as well as confusion and frustration in the team. However, in an informal interview, one of the physicians said that while it was certainly a good thing to improve documentation routines, this was not necessarily related to person-centred care.

This physician’s opinion was reaffirmed in a discussion held during the last 2 days of the programme, when the participants were using experiences gleaned from their learning group activities to develop new routines for the common care plan. In contrast to the inter-professional discussions about patients’ perspectives that took place during the overnight retreat, no suggestions for new ways to communicate based on dialogue came up during these final 2 days. Instead, words such as “blueprint”, “care plan” and “documentation” were bandied around and problems such as discontinuity in care and problems with work scheduling hindering person-centred care were raised again. This escalated into a heated discussion with the management leaders. Surprisingly though, the physician referred to above now claimed that the new care plan should enable them to handle even greater discontinuity in the team:

It shouldn’t matter if it is the same [person] or not. We’re developing a way of working that will allow us to divide our time because now we have a document [that follows the patient].

The ethnographer was astonished by this statement. So, she approached the participants in the learning group that had noted how they tended to take their own interpretation of the patient’s problem for granted, thereby overlooking the need to elicit the patient’s own perspective. She reminded them of this and then asked them what they thought about this faith in standard forms and the sudden silence about the importance of paying attention to the patient’s voice, but they fell silent. The RN then commented briefly that they had to develop “blueprints to follow”, and the physician added: “all the same, it’s documentation that we’ve got to have”. The NA listened quietly and then agreed with the ethnographer but was interrupted before she could elaborate. Several weeks after the programme had ended, the ethnographer posed the same question to one of the senior physicians who had participated. He paused and then said:

It will be a challenge to see that the care plan doesn’t become just one more piece of paper.

## Discussion: implications for future implementation programmes for person-centred care

In this article, we have described a dynamic and complex hospital context in which there are many professional boundaries, relationships and kinds of knowledge. All of these factors must be taken into consideration if the implementation of person-centred care is to become more than tentative (Harding et al. 2015). In this section, we discuss the success or failure of various aspects of the implementation process in order to elucidate what may be improved upon in future implementation programmes for person-centred care. We will focus on three factors: (1) the physicians’ resistance to implementation, (2) the silence about inter-professional communication and the implications of disciplinary knowledge for teamwork and a common care plan, and (3) the need to encourage participants to use the ethical insights they gain for developing a person-centred ethic in practice. These three factors were interlinked, and they could either facilitate or hinder learning and implementation of person-centred care. The following subsections further describe and comment on these factors.

## Understanding physicians’ resistance in context

It is often believed that it is difficult to get physicians involved in projects designed to achieve organizational change. However, their engagement is crucial since they occupy a powerful position within their organization (Baathe and Norbäck 2013). There is a tendency among managers in general, and managerial nurses and nurses in particular to blame the physicians for being resistant to change. To improve relations within the healthcare system it is important for all actors, including the physicians, to understand the reasons for this resistance. Studies of the contexts in which this resistance occurs have recently started to emerge (Snell et al. 2011; Chang and Ritchie 2015; Bååthe 2016). This study makes an important contribution to the literature by showing that contrary to the careers’ and managers’ preconceptions, the physicians did show a cautious interest in person-centred care.

In this study, it was noted that the physicians’ resistance did not primarily concern the concept of person-centred care. Instead, it was organizational factors that held them back—they had not been involved in making decisions about participation and their schedules were not adjusted to give them time to participate. Our study thus illustrates the problems of trying to bring about change if all of those affected are not involved from the start (Schein 2010). This had repercussions for collective learning about and implementation of person-centred care. This kind of problem is not unusual (Baathe 2015); physicians are seldom invited to influence the conditions that would allow them to be active in organizational improvement projects. There are several reasons for this. One is that management leaders tend to focus on the development of clinical skills while overlooking organizational and collective features of the profession (Snell et al. 2011). Biomedical epistemology focuses on “the solitary body of the individual sick person” and this can make it difficult to promote the importance of social relations for illness (Kleinman 1995: 37) and in the clinic. There needs to be greater awareness among the leadership of the way that these factors affect physicians’ reticence to participate in organizational improvement projects. Only then can they create better conditions for physicians to participate, and physicians may also enhance their professional self-awareness.

An important finding in this study was that the exclusion of physicians from decision making exacerbated their pre-existing mistrust of the hospital management leadership. We interpret the physicians’ silence when they met the clinic’s top management leader and their unofficial criticism of their own ward management leader as signs of insecurity at the clinic. Lack of psychological security tends to prevent dialogue and the creation of a learning organization (Edmondson 1999; Nembhard and Edmondson 2006). We see this as a serious hindrance to collective learning about and the implementation of person-centred care. The speech made by the top management leader revealed a discrepancy between the perspectives of the management and the physicians regarding the goals of care and organizational reality (Baathe and Norbäck 2013). Had they been aware of this discrepancy, the leader might have acted in a way that would enhance psychological security instead of reinforcing mistrust. Lack of appreciation of the different rationalities that guide those responsible for administration and economy from those who are clinically responsible for individual patients commonly leads to misunderstandings (Sager 2011). It is the responsibility of the managers to create awareness of and address these differing rationalities (Baathe and Norbäck 2013). We observed that the physicians’ resistance was met with interest by the facilitators but not by the managers. It is broadly recognized that the management leaders’ approach and ability to handle resistance to change with an attitude of curiosity and respect, and by adopting an inquiring rather than disapproving approach, is essential for implementation success (Loup 2005; Weiner 2009; Erichsen Andersson et al. 2018).

Altogether, understanding the physicians’ resistance in its wider context shifts the emphasis from laying blame upon them for being resistant to change to appreciating how organizational factors, such as management leadership, the different rationalities that exist in the clinic, the physicians’ area of expertise, and the structuring of the healthcare system in general give rise to these negative reactions.

## The silence about inter-professional communication and knowledge: implications for teamwork and a common care plan

The silence about inter-professional relations, professional boundaries and discipline-specific knowledge had implications for improving teamwork, creating a common care plan and developing person-centred care. Earlier studies of this particular implementation programme, with its stress upon documenting the patient’s narrative in partnership (Ekman et al. 2011), have described documentation as one of the greatest challenges to successful implementation (Britten et al. 2016). Our study confirms this finding but then also analyzes the factors that contribute to it.

Both the physicians and the nurses expressed anxiety about the implementation of person-centred care increasing their workload by demanding still more documentation. They devoted most of the time during the programme days to discussing documentation and, particularly, the division of labour between the RNs and the physicians. Documentation is already one of the physicians and nurses’ most important responsibilities, though each group is trained to document according to their particular professional expertise and they are each responsible for their “own” paperwork. Something that was not addressed in the programme was the fact that setting up care plans has traditionally been the responsibility of the RNs (Jansson 2010). Creating a *common* document and, furthermore, a common *care plan*, to which members of all the professional groups could contribute was thus innovative but also controversial and challenging. It evoked strong feelings about professional identity and disciplinary knowledge.

These feelings were also strengthened by the fact that professional identity and knowledge are related to hierarchy. In her research on care plans, Jansson (2010) notes that in traditional care plans, medical knowledge is generally superordinate to knowledge about caring. The RNs in our study spoke informally of how both the patients’ perspective and the caring perspective were overshadowed by the medical perspective in common care plans. Again, it is important to understand this in its broader context. Physicians are trained to be autonomous decision makers, and tend to place great value on this autonomy (Baathe and Norbäck 2013) and the organization of healthcare generally awards them the authority to define and solve problems in the treatment of patients (Wikström 2008).

This inequality in forms of knowledge needs to be acknowledged and addressed. Researchers who work on person-centered care agree that equal involvement by members of all the professional groups is necessary “to determine an optimal treatment plan based on the whole health condition of a patient” (Zhao et al. 2016: 4001, see also McCormack et al. 2010; McCormack and McCance 2010; Joynes 2018). However, inter-professional communication is generally characterized by unquestioned assumptions about which perspective is most important (Nilsen 2014). As long as these assumptions remain unquestioned and invisible, they continue to steer practice and work against the strengthening of inter-disciplinary teamwork (Edmondson 2012). It is important to note that the documentation process is consequently not a neutral task but is shaped by these assumptions about professional identity, boundaries and knowledge.

The overall lack of inter-professional exchange of ideas may be one of the reasons that it is so difficult to implement person-centred care in general. We therefore wish to stress the need for mechanisms to facilitate greater inter-professional dialogue. Space and opportunities should be provided to support managers and professionals in reflecting together upon their assumptions about professional identity, boundaries and knowledge (Erichsen Andersson et al. 2018) and to discuss their various perspectives on the goals of care and the organizational reality in which they all work (Baathe and Norbäck 2013).

## Healthcare workers’ need to learn person-centred ethics

The final factor we will discuss here is the lack of attention to developing a person-centred ethic during the implementation programme. Several of the problems with implementing person-centred care that were noted by the participants at the start of the programme were actually later addressed by them as the programme progressed: they trained in how to involve patients in care decisions, inter-professional communication, teamwork and the engagement of patients all improved. We observed how the participants’ mindsets altered during the programme; they became aware of how certain professional communication patterns may negatively impact upon the quality of care. For instance, they noted how they tended to conflate their own professional goals with those of their patients and realized that they needed instead to elicit their patients’ perspectives. Researchers have already pointed out that it is precisely the professionals’ appreciation of the difference between their own goals and those of their patients that is essential in delivering person-centred care (Britten et al. 2016). The physicians in our study found that gaining this insight helped them improve care as well as their medical treatment plans and this fuelled their motivation. This resonates with a recent study that showed that physicians are more likely to engage in creating organizational change if they see that it can enhance their sense of professional fulfilment, such as by strengthening the grounds for medical decision making (Lindgren et al. 2013). Consequently, learning about person-centred care and ethics was initiated during this implementation programme.

Unfortunately, though, as the programme progressed, these ethical issues tended to become overshadowed by other matters and ultimately, they were eclipsed altogether by discussions about documentation, routines and division of labour. Earlier research on this implementation programme has also noted that there was no enduring change of mindset among participants to a person-centred care approach (Britten et al. 2016). Again, our study affirms this finding but also puts it in context and identifies the factors that lead to such an outcome.

We found that the structure of the programme did not support the participants in putting what they had learned about person-centred care as an ethic into practice. We propose that they would have required more guidance in internalizing these insights and then integrating them into the common care plans as part of their everyday work. It is possible that the facilitators overlooked the cultural context or overestimated the participants’ ability to put ethical insights into practice on their own.

However, we believe that a major reason that these insights were not then reflected in altered practice was the tension between different epistemological viewpoints about how change is brought about. Following the principles of action learning, the facilitators aimed to create opportunities for an iterative collective learning process. By contrast, the management leaders saw person-centred care as a systematic method that could be practised by individual carers and be monitored and measured (see Table 1 for the steering committee’s goals). This is a continuation from earlier implementation projects conducted at this medical emergency ward, as described above in the first section on findings. Typically, these earlier projects followed a cognitive, linear and instrumental planning rationality. Bååthe (2016) and Nilsen (2015) note that it is common for healthcare managers to mechanize and put what has been learned into manuals instead of internalizing it, and this may in fact work against genuine learning (Schein 1999 referred to in Grill et al. 2015: 449). Also, broader contextual factors tended to reinforce this task-oriented approach since healthcare policies generally reflect a heavy reliance on methods and tasks rather than offering guidance on ethics and how to handle the complexity and dilemmas involved in care (Schuster 2006).

Like the Health Foundation (2014), we contend that person-centred care is not simply a model that can be applied but is a philosophy of care whose implementation requires structural and organizational adaptation and strong leadership. It is therefore important for the facilitators of implementation programmes to first and foremost involve management leaders in learning about iterative collective learning processes (Erichsen Andersson et al. 2015) and increase awareness of the relationship between ethics and methods (Schuster 2006).

### Strengths and limitations

One strength of this study was the ethnographer’s longstanding relationship with the care staff and their managers. This made it possible for her to participate in the more delicate situations that processes of change often give rise to. Her familiarity with and prior ethnographic knowledge of the ward under study also enabled her to see this implementation programme in its broader context. Another important strength was the intellectual co-production between the co-authors and their diverse perspectives. However, we need to bear in mind that there were also methodological limitations to the study that should be considered when interpreting the results. One is that a single observer did all the observations. Within social anthropology there is a long tradition of researchers doing fieldwork alone, and the ethnographer in this study is a trained anthropologist. Still, it would have been valuable to be able to compare the initiated ethnographer’s observations with the observations of yet another observer who was not familiar with this particular hospital context in order to make up for eventual biases. The close co-operation with co-authors who contributed with alternative interpretations and perspectives was an important way to counteract this. It would also have been valuable to complement the data with formal interviews. Still, the continuous informal interviews and natural conversations with the participants made it possible to cover a wide variety of voices, perspectives and experiences in the group. There were other limitations to the study; specifically, the data collection was limited to a single site. However, our thick and detailed description of local contextual factors should help our audience to transfer insights gleaned from this setting to others. Similarly, the hospital organization described here, with its tension between hospital management and healthcare professions in the current era of corporate managerialism on one side, and the more traditional struggle between various healthcare professions and disciplinary knowledge on the other, may be recognized in many healthcare settings in the world. It could furthermore be argued that a single case study format of research can provide an opportunity to gain deep knowledge, and allow for explanation of the phenomena observed, and thus contribute towards transferable scientific knowledge.

## Conclusion

Our study shows three important, previously neglected aspects of healthcare providers’ learning processes emerging in the dynamic context of an innovation in a complex environment. First of all, resistance of change is both a result of leadership and management of learning in the implementation process *and* a necessary condition for learning and implementation of the innovation into daily practice. Thus, both leadership and the managing of complex innovations to improve daily practices, such as person-centred care could be seen as a process of, as well as a condition for learning. Consequently, learning between actors (here foremost managers and physicians) is a dynamic and iterative process, and it unfolds in a recursive relationship between daily practice and structural, cultural and normative relations. This means that the processes of negotiating and (re-)constructing daily practice is an interactive dynamic process that depends on the feasibility of the innovated daily practice. If it is integrated, it may then be re-negotiated in the social system (into the social norms and relations of involved actors). Importantly, the study shows that the leadership and the management of person-centred care need to focus both on involvement (social and structural relations with strong professionals), and dialogue about management prioritization related to general policies and best practice in the clinic (cultural and normative relations). Involvement is an important structural aspect in the leadership and management of learning process in efforts to improve daily practice. Dialogue about priorities is an important feature of good leadership in order to connect implementation and learning to the cultural norms of the clinic’s everyday practices.

Secondly, documentation as practice develops in a recursive relationship between practice and learning. The documentation practice could be seen as a materialization of learning processes concerning the content of work at the clinic, labour division and the inter-professional relations. Thus, documentation practice drives and constitutes change in daily organization of work in the clinic. A dialogue on documentation and the common care plan is then a key aspect of the learning process and the implementation of person-centred care. Significantly, as documentation according to this implementation programme is essential for safeguarding person-centred care, facilitators and the implementation structure must support the healthcare providers in a profound reflection on professional boundaries, disciplinary knowledge and power relations in the team.

Thirdly, the study also illustrates that the clinical mindset and ethical insights, i.e. normative relations and cultural aspects embedded in the documentation practice, are key aspects in the learning process. The ability to practice learned normative aspects and ethical insights is necessary for the implementation of this complex innovation. To approach learning and implementation of innovation as an iterative and dynamic process that is related to social norms and relations between involved actors from the preparation and planning stage creates the grounds for sustainability and legitimization of the new practice of person-centred care.
